# Menstrual Hygiene Problems and Challenges Faced by Adolescent Females in Rural Areas: A Narrative Review

**DOI:** 10.7759/cureus.40438

**Published:** 2023-06-14

**Authors:** Vijiya Kashyap, Sonali G Choudhari

**Affiliations:** 1 Public Health, School of Epidemiology and Public Health, Datta Meghe Institute of Higher Education and Research, Wardha, IND; 2 Community Medicine, School of Epidemiology and Public Health, Jawaharlal Nehru Medical College, Datta Meghe Institute of Higher Education and Research, Wardha, IND

**Keywords:** menstrual waste consequences, menstrual waste incineration, menstrual hygiene products, adolescent health, menstrual hygiene practice, menstruation

## Abstract

Menstruation is a vital sign of reproductive health and development. Menstrual hygiene practices are consequently a significant public health issue. However, menstrual practices are still tainted by taboos and other sociocultural constraints, which adversely affect health since adolescent females in rural areas are unaware of the scientific facts regarding menstrual hygiene practices. The Indian government has recognized the significance of menstrual hygiene and developed and implemented several programs and schemes for menstrual hygiene management (MHM). But due to a lack of, little, or inaccurate knowledge and cultural practices and socio-economic status, adolescent females face many obstacles and suffer from health issues. A comprehensive literature and data search was done using key databases such as PubMed and Google Scholar and other sources such as the Ministry of Health and Family Welfare (MoHFW), the United Nations International Children's Emergency Fund (UNICEF), the World Health Organization (WHO), and Google to identify the relevant articles and reviewed publications using full-text search. A total of 40 articles out of 1,461 were selected for review after the screening and elimination of repeated articles. The objective of this literature review is to assess the awareness and knowledge of the importance of menstruation, the understanding of safe menstruation practices, the significance of properly disposing of menstrual products, and the knowledge of how to guard against reproductive system infection and its consequences and also to identify the problems and challenges faced by adolescent females during their menstrual hygiene practices or management. The core of many health issues is misinformation, myths, erroneous beliefs, lack of awareness, and incomplete or incorrect knowledge about menstruation. Therefore, it is essential to teach adolescent females about hygienic behavior and safe menstrual practices.

## Introduction and background

The World Health Organization (WHO) defines "adolescents" as individuals in the 10-19 years age group, the period between childhood and adulthood [1]. It is a particular stage of human growth and development and is essential for laying the foundation for long-term health. They grow in terms of their physical, mental, and emotional development. This affects their feelings, ideas, choices, and interactions with other people. Currently, an estimated 600 million females worldwide are between the ages of 10 and 19, and approximately 90% of these females live in developing countries. Females in this age group are in a crucial stage of development where they go through life-changing experiences, and these lay the groundwork for the majority of the rest of their lives [2].

Menstruation is described as cyclical bleeding from the uterine corpus that takes place between menarche and menopause. One of the most significant changes that females go through is menarche, the start of menstruation [3]. The first menstrual cycle in the adolescent's life is called menarche. Menarche can begin as early as age 10 or as late as age 16, although it commonly begins between the ages of 12 and 13. In India, the menstrual age spans from 10 to 16 years (the average age is 13.5 years) [4]. In society, menstruation is typically viewed as impure. Because of the stigmatization of menstruation of females and the restrictions placed on them in the household, a negative attitude toward this phenomenon has been concealed [5]. Many menstrual ideas and perspectives, which either oppose or support the health of adolescent females, may be found worldwide. Studies have revealed that erroneous knowledge of the menstrual cycle, menstrual hygiene, and self-care practices is less widespread than superstitions, illogical beliefs, and misunderstandings [6]. Adolescent females in rural settings have been concerned about menstrual hygiene management (MHM) practices [7]. The ability to manage menstruation hygienically and respectfully is a primary priority for females, as are the tools, resources, and surroundings required [8]. Adolescent females who are more informed about menstrual hygiene and safe practices are less likely to get vaginal infections and the consequences that come with them. Menstruation is a normal occurrence, but it is also linked to various attitudes and practices that occasionally have a detrimental effect on one's health. In order to better understand the knowledge, sanitary condition, and menstrual practices of adolescent females in rural areas, a review study was done [9].

To avoid any embarrassment brought on by menstrual leakage, some parents advise their daughters to remain at home until they have stopped menstruation. "During menstruation, the mother may advise her daughter to stay at home to avoid humiliation and also to save them from ruining their school clothes. It is preferable to keep them at home until their periods are finished because they have no space to go to clean their cloth and change in their school" (K-II traditional leader). Although females are educated about marriage at menarche, female guardians asserted that the focus was on their need to refrain from "playing," which is another word for having sex, as this would put them at risk of conceiving and backing out of school [10]. There were prohibitions on bathing and a taboo against burying menstruation fabric that had been stained in various areas of the country. Clothes should be cleaned before being buried or reused. In order to prevent others from seeing them, washing and drying are often done in private or in a hidden location [11]. It has been demonstrated that unhygienic menstrual practices might contribute to reproductive tract infections, cervical cancer, leaving or dropping out from school, low self-esteem, a bad quality of life, and unsatisfactory academic achievement [12].

Understanding menstrual practices, menstrual products, and disposal ways was the goal of this review article. The needs and management of menstrual hygiene are covered, as well as a summary of the existing knowledge analysis.

## Review

**Figure 1:**
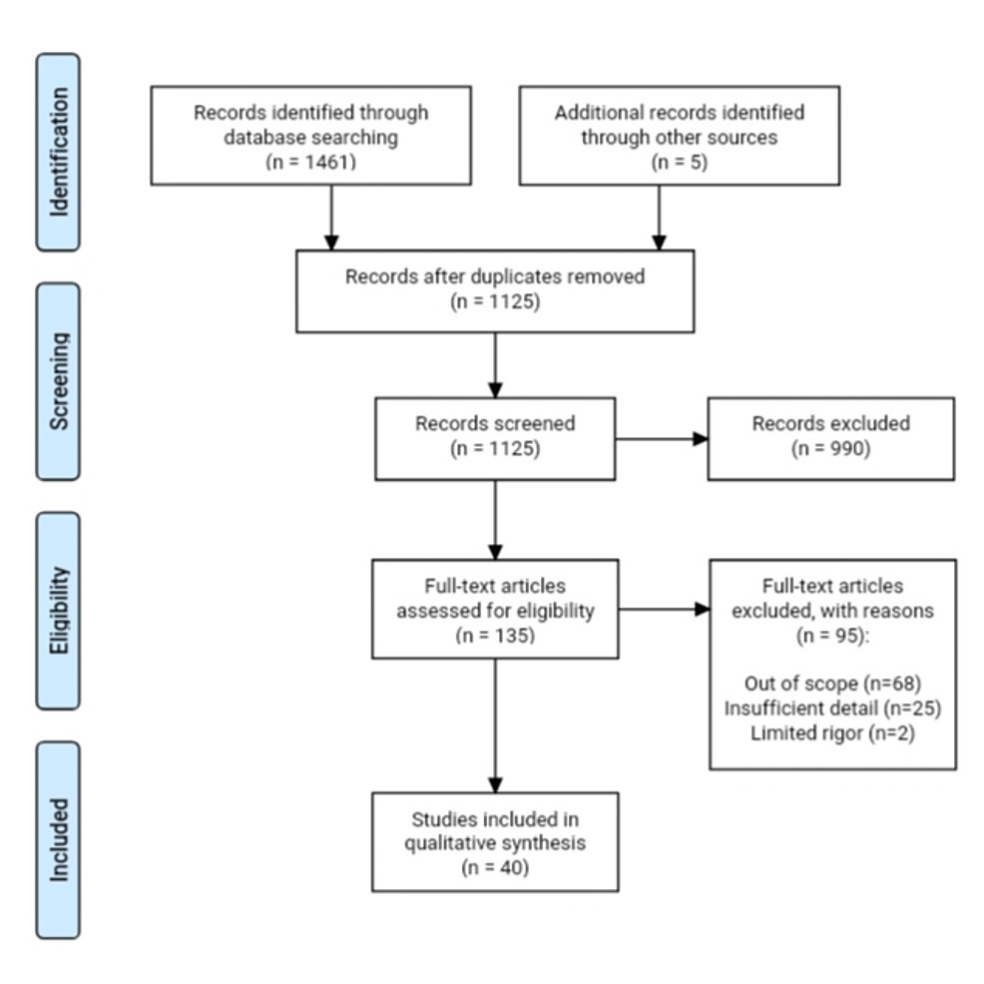
Inclusion and exclusion criteria of this study.

This narrative review focuses on challenges and problems faced by adolescent females in rural areas while practicing menstrual hygiene. The database searched includes PubMed and Google Scholar, as well as other sources such as the Ministry of Health and Family Welfare (MoHFW), the United Nations International Children's Emergency Fund (UNICEF), the World Health Organization (WHO), and Google, to identify the relevant articles and reviewed publications using full-text search. Several keywords used to find relevant articles included the following: menstruation, menstrual hygiene, adolescent health, menstrual practice, menstrual hygiene products, and menstrual waste incineration. Mostly, articles from 2017 to 2022 were taken. The excluded articles were those with adolescent females from urban areas or with any disabilities and also those articles that are not available in the English language, which is depicted in Figure 1.

Discussion

**Figure 2:**
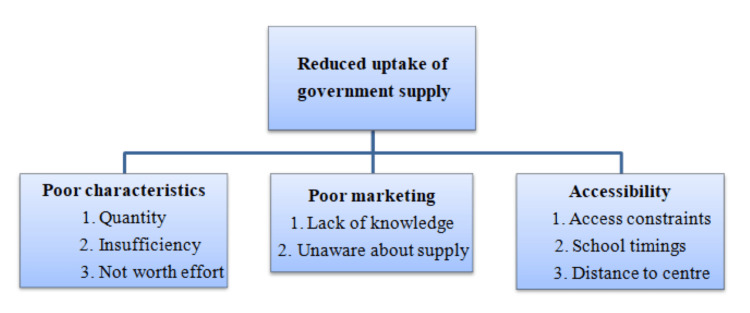
Reduced uptake of government supply.

Almost all teenage females who were menstruating used sanitary pads, according to a review of literature from adolescent females in a rural Puducherry area. The target population did not use the government product as widely despite having a platform for promoting teenage menstruation practices. There were not enough pads provided to complete a cycle, and this served as a curb in surrounding clinics. For those who used government-supplied pads, the quality of pads was a problem (Figure 2) [13].

About two-thirds of rural school females of Ugandan reported missing class at least once each month due to menstruation, according to a survey. Only 54% of Indian females reported attending class while having their periods [14]. Since standing to answer questions is a typical practice in many schools and writing on the board in front of the class may disclose menstrual stains, leaks, or odors, females who frequently attend school while menstruating report feeling distracted, unable to concentrate, and reluctant to participate [15].

According to several studies, the unawareness and inadequacy of preparedness about puberty and menstruation raise myths and leave females vulnerable to low self-esteem and feelings of shame [16]. Families Matter, which is a wide-scale program [17], has demonstrated that through the government health and education systems and the use of evidence-based curriculum on health and sexual guidance, interventions that improve parent-child sexuality communication can be successfully implemented on a large scale. These programs might improve the general public's awareness of reproductive health. These programs might be expanded to include puberty education, which could aid these young females who lack information and understanding of their own bodies [18].

Menstrual Products

Arunachalam Muruganantham, also known as the "Padman," created the affordable, environmentally friendly sanitary napkin that has helped countless females in rural India. After watching his wife indulge in unclean behavior while she was menstruating, he had the idea to create this device. When selecting menstrual products, convenience, price, safety, females' dignity, and environmental sustainability must all be taken into account [19]. Throughout the menstrual cycle, various absorbents have been employed, which are mentioned in Table 1. The absorbents made up of fabric are reusable. Prior to the following use, they must be cleaned and dried in the sun. Plastic and cellulose are the main components of disposable sanitary pads. The biodegradable, environmentally sustainable sanitary napkins are made of water hyacinth, banana, bamboo fiber pads, and sea sponges, but they are not readily available. Tampons are available in both reusable and nonreusable varieties. Also, menstrual cups are utilized. These must be placed inside the vagina. Consequently, they are not appropriate for all adolescent [20].

**Table 1:** Different types of menstrual products. UTIs: urinary tract infections

Menstrual products	Advantages	Disadvantages
Washable cloth pad [21]	Reusable and environmental friendly	Cloth menstrual pads need to be washed with soap, thoroughly dried, and maintained. Pads can cause reinfection if not sterilized
Commercial sanitary pad [22]	Easily available, convenient to use, and has fewer chances of infection	Cotton is not used in all pads. Some pads are made of synthetic materials and contain chemicals, which may be prone to several health issues, expensive, and not very eco-friendly
Menstrual cup [23]	Eco-friendly lasts for a long time and reduces the risk of getting a bacterial infection, such as toxic shock syndrome (TSS)	Increased chances of vaginal infections might be caused if proper precautions and directions are not followed
Tampons [24]	Comfortable and convenient to use	Not eco-friendly and has a higher risk of TSS rarely but can leave a residue in the vagina, resulting in bacterial infections and inflammation
Banana fiber pads (sold under the brand name Saathi) [25]	Biodegradable, less expensive, and specially designed for rural areas to handle health issues	They can cause rashes and UTIs because they are not much skin-friendly
Sea sponge pads [26]	Reusable for up to six months, biodegradable, and less expensive	They can break apart while inserting or removing
Water hyacinth pads (sold under the brand name Jani) [27]	Low-cost, eco-friendly, and biodegradable	Less risk of TSS. The lack of awareness

Menstrual Practice Consequences

Females in rural regions are unlikely to have access to and/or use menstrual products to manage their periods in addition to frequently lacking access to soap, clean water, safe bathrooms, and functional latrines with trash disposal facilities. These issues have an immediate and lasting effect on females' self-worth and abilities, as well as their ability to engage in regular activities such as education and employment and maintain good health [28].

Unhygienic menstrual hygiene management (MHM) practices could lead to opportunistic infections such as *Candida* due to abnormally wet conditions in the vulvovaginal area. Without thorough cleaning and drying, it may be difficult to remove *Candida* from clothes after they are contaminated [29]. There was a 2.3 times greater likelihood of urogenital infection symptoms in people who used reusable pads. A case-control research by Das et al. in Odisha, India, discovered that females who used reusable pads were 2.8 times more potent to be diagnosed with any of these urogenital infections, such as urinary tract infection (UTI) or bacterial vaginosis (BV), than those who did not [30]. A study reported that a significant ratio of females feels ashamed in buying pads, signifying the high prevalence of stigma associated with menstrual pads, a misconception that contributed to lowering their self-efficacy in achieving healthy MHM [31].

Sanitation systems cannot handle the menstrual absorption materials because they were created with urine and feces in mind. Due to their inability to move through the sewer pipes, these absorption materials clog them, resulting in system backflow and a major health risk. Menstrual waste is dumped into water bodies by residents who live along riverbanks, polluting them. Sanitary products that have been soaked in the blood of an infected adolescent female or adult female may contain HIV viruses or hepatitis, which can persist in soil for up to six months [11].

Menstrual Hygiene Management

There are several ways to inform and educate people about menstruation, including by providing them with thorough sexual education that is appropriate for their environment. With the use of this knowledge, people will be able to recognize menstruation as a typical biological occurrence and acknowledge it, resulting in hygienic procedures, such as cleansing the genital area, washing hands with a disinfectant before and after using absorbents, and replacing absorbent goods three to four times every day [32]. When conducting interventions, it is crucial to inform the public about the importance of MHM and teach adolescent females how to utilize the menstrual products provided in humanitarian operations [33].

Menstrual waste disposal can be managed using incineration, which reduces volume and pathogens while also reducing trash, hence reducing environmental issues brought on by inappropriate disposal. As part of its Swachh Bharat (Clean India) initiative, the Indian government has encouraged the use of incineration for menstrual waste (2018). With an estimated 121 million females disposing of 12.3 billion pads yearly, India has a very high volume of menstrual trash, amounting to 113,000 tonnes [34]. Along with her husband, Swati Bedekar, a member of the Vatsalya Foundation (Gujarat), created a clay incinerator for the disposal of used sanitary towels. The name "Ashudhinashak," which in Sanskrit means "annihilation of impurities," was given to the incinerator. It is possible to nourish plants by combining the created ash with soil. This groundbreaking invention reduces trash and enables females to dispose of their old napkins in an environmentally responsible manner [35].

Mobile phone messaging services have been utilized in numerous community health projects in India (Mobile Vani, Kilkari, and Mobile Academy), as well as other countries, to broadcast health information and enhance the delivery of services and behaviors that promote health. The success of these projects shows that it is possible to harness the widespread usage of mobile phones in rural India to convey the right information about menstrual hygiene practices and increase awareness among adolescent females [36]. Now, menstrual health is considered a public health concern that is closely connected to a number of civil rights and the achievement of the Sustainable Development Goals (SDG), thanks to persistent and innovative sponsorship by a variety of shareholders and platforms, alliances, and networks at the international, regional, and national levels. As a result, government agencies and ministries (such as those in the areas of education, health, gender, and water, sanitation, and hygiene {WASH}) are more willing and ready to act [37].

Interventions and Schemes

The UNICEF and WHO recommended installing WASH, or water, sanitation, and hygiene, facilities in schools [38]. Every school in India has been equipped with WASH facilities, including soap and water for sanitation and private areas for menstrual-absorbent change and disposal, as part of the Swachh Bharat: Swachh Vidyalaya initiative. The Ministry of Health and Family Welfare has started a campaign to encourage menstrual hygiene among teenage females between the ages of 10 and 19 in rural areas. Rural adolescent females were provided with a bag of six sanitary napkins known as "Freedays" for Rs 6 for each pack of six napkins as part of the initiative, which was initially introduced in 2011 in 107 selected districts across 17 states [39]. A free sanitary pad program has been in place in Tamil Nadu since 2011 for females living in rural regions. Additionally, they are eligible to get three packs of pads every two months, as well as iron supplements and information about menstruation from an "anganwadi" (female community health worker). Maharashtra and Chhattisgarh have similar programs but are less established [40].

## Conclusions

Menstrual hygiene should be recognized as a major public health issue as it is a leading cause of serious health issues in growing adolescent females and is also indirectly responsible for environmental hazards. So, they should be aware of the consequences of ignorance of safe menstrual hygiene practices. Menstrual hygiene can be promoted by delivering proper knowledge regarding safe practices, the advantages of using biodegradable sanitary products, properly disposing of menstrual waste products, and the importance of menstrual waste management. Teachers can play a major role in imparting menstrual hygiene education not only to females but also to males so they also understand their roles and responsibilities and can help in changing the mindset or false perception of the societies or communities who thought menstruation was taboo and impure. Nowadays, technologies (Kilkari and Mobile Vani) can also act as an intervention in delivering knowledge on a public health issue. Many programs and schemes are implemented by the Ministry of Health and Family Welfare, such as the offering of sanitary napkins at low cost in rural areas, the installation of incinerators for disposable menstrual waste products, and the distribution of iron tablets in rural areas of Tamil Nadu. The WHO and UNICEF recommended having WASH facilities in schools. The core of many health issues is misinformation, myths, erroneous beliefs, the lack of awareness, and incomplete or incorrect knowledge about menstruation. Therefore, it is essential to teach adolescent females about hygienic behavior and safe menstrual practices.
